# A Comparative Study of Clinical, Hematological, and Biochemical Profiles in Eosinophilic and Non-eosinophilic Phenotypes of Chronic Obstructive Pulmonary Disease (COPD) During Acute Exacerbations

**DOI:** 10.7759/cureus.92329

**Published:** 2025-09-14

**Authors:** Sudheer Yadav, Bechan Kumar Gautam, Ajay Kumar, Akhilesh K Dubey

**Affiliations:** 1 Medicine, Baba Raghav Das Medical College, Gorakhpur, IND

**Keywords:** biomarkers, copd, eosinophils, exacerbation, inflammatory phenotypes, inhaled corticosteroids

## Abstract

Chronic obstructive pulmonary disease (COPD) continues to be a major global health concern, with emerging evidence suggesting that blood eosinophils may help distinguish between inflammatory subtypes during acute exacerbations. This observational cross-sectional study conducted at Baba Raghav Das (BRD) Medical College, Gorakhpur examined 60 patients with acute COPD exacerbations, categorized into eosinophilic (>300 cells/μL, n=20, 33.1%) and non-eosinophilic (≤300 cells/μL, n=40, 66.9%) phenotypes based on peripheral blood eosinophil counts. Non-eosinophilic patients demonstrated significantly higher systemic inflammation markers, including C-reactive protein levels (17.35 ± 3.8 vs 8.2 ± 5.3 mg/L, p<0.001), neutrophil percentages (79.5 ± 3.5% vs 66.2 ± 4.7%, p<0.001), and hepatic enzymes, along with longer hospital stays (6 vs 4 days, p=0.002). Eosinophilic patients were more likely to receive inhaled corticosteroids (66.0% vs 44.4%), while those with very low eosinophil counts (<150 cells/μL) more frequently received systemic corticosteroids (46.7% vs 19.1%, p=0.023). These findings suggest distinct inflammatory profiles between COPD phenotypes during acute exacerbations and support the potential utility of eosinophil-guided therapeutic approaches, though prospective studies are needed to confirm these associations and their clinical implications.

## Introduction

Chronic obstructive pulmonary disease (COPD) is an incurable progressive lung disease and is now considered the third leading cause of death in the world [1]. It represents a major health problem internationally. It is mostly a combination of chronic bronchitis and emphysema. These disease states make it difficult to breathe and acquire oxygen, and they increase the likelihood of death. In essence, COPD is a result of persistent, multi-system, and complex inflammation. COPD stems from complex and multi-system inflammation, which is ongoing and leads to permanent damage and structural alteration of the airways, lung tissue, and blood vessels, in addition to the hallmark difficulty in breathing associated with the disease [2-4].

Lung capacity reaches its peak between the mid-30s to early-40s in humans. After this peak is reached, a natural decline is initiated. For sufferers of COPD, this decline is exacerbated by acute exacerbations of flare-ups. Acute exacerbations are defined as acute episodes in which a person's breathing symptoms, such as breathlessness, coughing, and expectoration of phlegm, manifest and worsen considerably [5]. These episodes manifest as a result of chronic bronchitis, emphysema, and inflammation, which cause long-term damage to the airways and blood vessels. For the last 30 years, focus has been placed on understanding the flare-ups, and this has been a focus of research due to the lack of understanding regarding flare-ups and their impact on poor patient health [6]. Understanding exacerbations is critical, as their frequency and severity are directly proportional to the rate of progression of the disease, quality of life, and overall healthcare spending [7].

An example of a key biomarker that has appeared recently is a noninvasive blood test known as eosinophil count. It enables physicians to differentiate between different inflammatory subtypes of COPD, supporting more tailored therapeutic approaches. At a primary care level, eosinophil count is easily accessible and, therefore, can be incorporated into the routine care of patients with COPD. Patients with higher eosinophil counts not only show a greater risk of exacerbation [7], but also a more pronounced response to inhaled corticosteroids [8,9]. Conversely, low eosinophil counts may be indicative of a different disease form that, if not treated, may lead to mismanagement and unnecessary therapeutic interventions. This has resulted in a change of medical paradigm from a one-size-fits-all approach in COPD towards more phenotyping [10]. It is now recognized that there are several different subgroups of patients with COPD, who are likely to have different underlying etiologies, manifestations, and therapeutic responses. COPD is further subclassified as eosinophilic, or eosinophil-dominated inflammation, which is hypothesized to have some form of distinct but not fully understood inflammatory process [11]. Understanding these subtypes enables tailored therapeutic strategies that are the foundation of precision medicine in the management of COPD.

Some researchers suggest that patients with eosinophilic COPD may have distinctive clinical features such as differing patterns of exacerbations, responses to treatment, and prognosis in contrast to those with no or low eosinophil counts [12,13]. However, in-depth studies that compare the clinical, metabolic, and inflammatory profiling of these patients during exacerbations are surprisingly sparse. Although emerging evidence suggests that patients with eosinophilic COPD may have distinctive clinical features such as differing patterns of exacerbations, responses to treatment, and prognosis compared to those with low eosinophil counts, comprehensive comparative studies examining the clinical, metabolic, and inflammatory profiles during acute exacerbations remain limited. This study aimed to address this knowledge gap by comparing the clinical features, hematological parameters, and biochemical profiles between eosinophilic and non-eosinophilic COPD patients during acute exacerbations, with the goal of informing more precise therapeutic approaches.

## Materials and methods

Study design

The study was carried out in the Medicine Department at Baba Raghav Das (BRD) Medical College, Gorakhpur, where the focus was on the comparative clinical, hematological, and biochemical assessment of eosinophilic and non-eosinophilic subtypes of COPD during acute exacerbations. This approach was aimed at capturing the microcosm of the differences that exist in these subgroups during exacerbations.

Study population

The study was conducted for one year or until the sample size was achieved, whichever came first. Participants in the study were recruited from outpatient and inpatient facilities to adequately capture all cases of COPD in acute episodes. There was a careful screening of all eligible patients coming to outpatient or emergency departments and requiring admission for exacerbation.

Sample size and statistical considerations

This study utilized convenience sampling over a one-year period to achieve the target sample size of 60 patients. While formal power calculations were not performed a priori, this represents a limitation that should be considered when interpreting results. The sample size was deemed adequate for exploratory analysis of phenotypic differences.

Inclusion and exclusion criteria

Eligible individuals ranged from 40 to 90 years old and had confirmed COPD based on a post-bronchodilator FEV1/FVC ratio under 0.7, the established standard for assessing pulmonary function. They were also required to possess at least 10 pack-years of smoking exposure, recognized as the key contributor to COPD in this demographic.

Those with concurrent asthma were barred from participation, since it could skew interpretations of eosinophil counts and inflammation indicators. Further disqualifications encompassed current malignancies, ischemic heart conditions, heart failure, or alternative long-term pulmonary disorders that might affect research variables or conclusions. Such protocols promoted a consistent cohort and lessened possible distortions in the data.

Methodology

Upon hospitalization for acute COPD exacerbation, patients underwent comprehensive clinical assessment within six hours of admission. Blood samples for complete blood count (including eosinophil count), inflammatory markers (C-reactive protein, ESR), and biochemical parameters (hepatic and renal function) were collected within 24 hours of admission and before initiation of systemic corticosteroid therapy when possible. Arterial blood gas analysis was performed to assess respiratory function and acid-base status.

Patients were stratified based on peripheral blood eosinophil concentrations into eosinophilic (>300 cells/μL) and non-eosinophilic (≤300 cells/μL) groups, consistent with established cutoffs used in previous COPD phenotyping studies. Demographic data, medical history, smoking exposure, COPD duration, and previous exacerbation frequency were systematically documented. Physical examination focused on respiratory system assessment and signs of acute exacerbation severity.

Data analysis

Information was inputted and refined via IBM SPSS Statistics for Windows, Version 30 (Released 2024; IBM Corp., Armonk, New York, United States). Summary statistics encompassed averages ± standard deviations for numerical variables and counts/percentages for categorical ones. Intergroup variations were assessed through unpaired t-tests for numerical data and chi-square tests for relevant categorical data. Relationships involving clinical and lab metrics with eosinophil concentrations were evaluated using binary logistic regression, with emphasis on forecasting respiratory failure and treatment results. Significance was defined by p < 0.05, incorporating suitable confidence intervals.

Ethical considerations

The study received full approval from Baba Raghav Das (BRD) Medical College's Institutional Ethics Committee, adhering to human research standards. All participants provided written informed consent after detailed explanations of procedures, goals, risks, and benefits, highlighting voluntary involvement and data confidentiality. Information was managed securely, accessible only to approved staff, with identifiers stripped for anonymity in analysis and reports. The research followed the Declaration of Helsinki and Good Clinical Practice principles, with ongoing ethical oversight.

## Results

A total of 60 individuals experiencing acute COPD flare-ups were recruited for the research, yielding a robust collection of information for evaluating differences between eosinophilic and non-eosinophilic subtypes. Patient breakdown indicated that non-eosinophilic cases dominated at 66.9% (n=40), with eosinophilic cases comprising 33.1% (n=20), highlighting the higher occurrence of the non-eosinophilic variant among participants.

Examination of demographic characteristics uncovered notable trends between the subtypes, as detailed in Table 1. Age profiles showed that the bulk of individuals in each category fell within the 50-70-year range, though those with eosinophilic features were marginally younger, averaging 57.81 ± 13.96 years, versus 62.94 ± 15.48 years for the non-eosinophilic group. In terms of sex, male patients were more common in the non-eosinophilic cohort (60.0%), whereas female patients edged out slightly in the eosinophilic cohort (51.06%). Assessment of tobacco use revealed higher rates among non-eosinophilic participants (33.7%) than eosinophilic ones (17.0%), yet the broader sample was largely composed of non-smokers, pointing to alternative contributors like pollution or occupational hazards in COPD onset.

**Table 1:** Distribution of cases based on absolute eosinophil count (AEC) levels according to age, gender, and smoking status Data presented as number (percentage) for categorical variables and mean ± standard deviation for continuous variables.

Sociodemographic Data		Non-eosinophilic (≤300 cells/µL)		Eosinophilic (>300 cells/µL)	
		N	%	N	%
Age (years)	≤50 years	9	9.47	8	17.02
	50–70 years	62	65.26	34	72.34
	>70 years	24	25.26	5	10.64
	Mean ± SD	62.94 ± 15.48		57.81 ± 13.96	
Gender	Male	57	60.00	23	48.94
	Female	38	40.00	24	51.06
Smoking	Yes	32	33.70	8	17.00
	No	63	66.30	39	83.00

**Figure 1:**
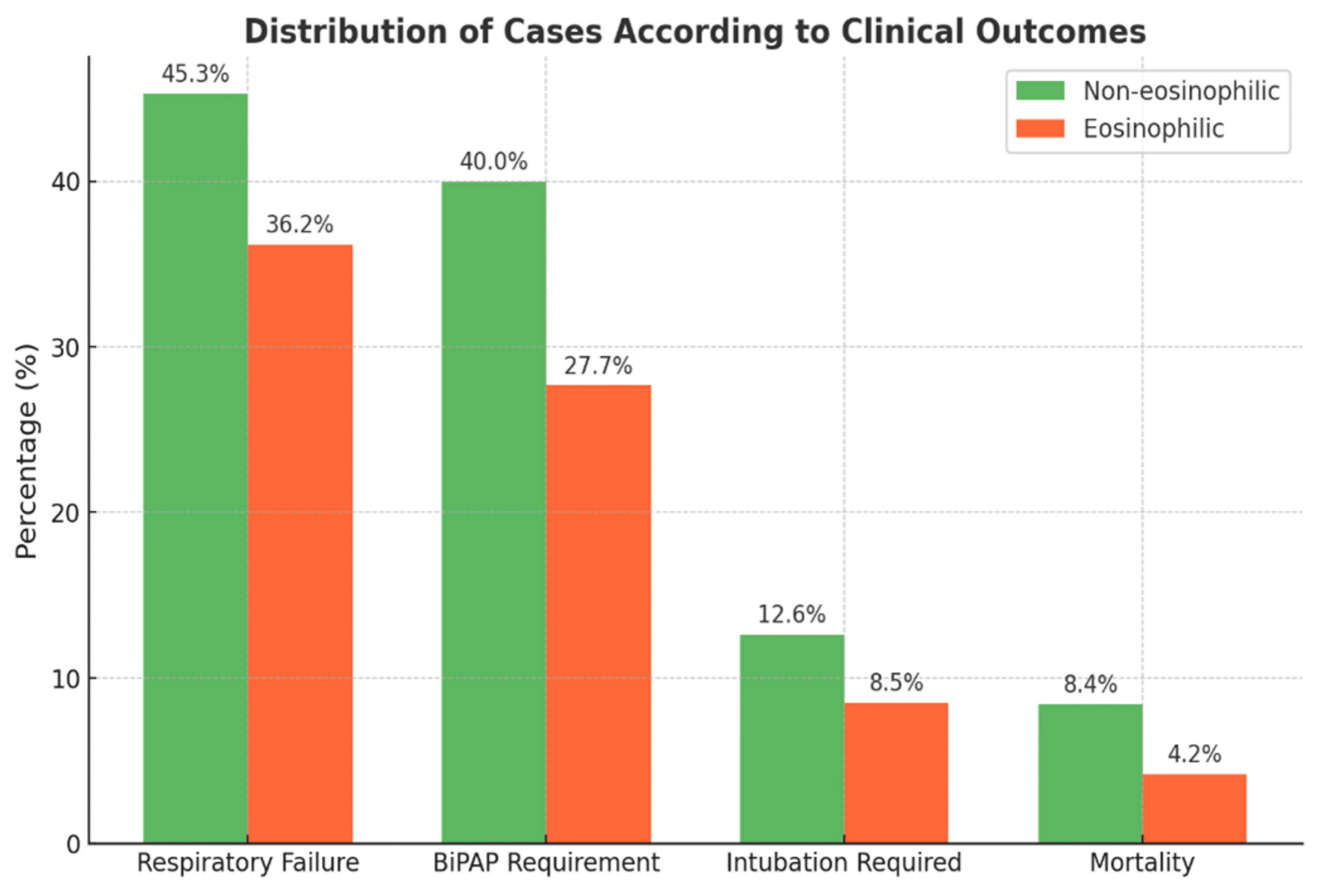
Distribution of cases based on signs and symptoms (N = 60, %) Data presented as the percentage of patients in each phenotype group. BiPAP: Bilevel positive airway pressure

Clinical presentation analysis revealed that shortness of breath was universally present in both groups, reflecting the fundamental nature of this symptom in COPD exacerbations. However, cough and sputum production showed significant variations between the phenotypes, being more commonly observed in non-eosinophilic patients, as illustrated in Figure 1. This finding suggests different underlying inflammatory mechanisms and pathophysiological processes between the two phenotypes.

Evaluation of lab metrics uncovered notable variations across the subtypes, detailed in Table 2. Individuals with non-eosinophilic traits showed considerably increased concentrations of various inflammation and biochemical indicators relative to those with eosinophilic traits. Notably, CRP values were substantially higher in the non-eosinophilic cohort (17.35 ± 3.8 mg/L) than in the eosinophilic one (8.2 ± 5.3 mg/L), yielding a p-value of <0.001 (t = 8.42). Such a pronounced disparity points to intensified widespread inflammation among non-eosinophilic cases amid acute episodes.

**Table 2:** Distribution of cases based on laboratory parameters of AECOPD patients (N = 60, mean ± SD) TLC: Total leucocyte count; AECOPD: acute exacerbation of chronic obstructive pulmonary disease; SGOT: serum glutamic-oxaloacetic transaminase; SGPT: serum glutamate pyruvate transaminase

Laboratory Parameters	Non-eosinophilic (AEC ≤300 cells/μL) (n=95)	Eosinophilic (AEC >300 cells/μL) (n=47)	p-value
Hb	12.62 ± 2.1	12.80 ± 2.3	0.642
TLC	15454.9 ± 1933.1	12500.4 ± 5275	0.306
CRP	17.35 ± 3.8	8.2 ± 5.3	<0.001
S. creatinine (mg/dl)	1.02 ± 0.50	0.97 ± 0.37	0.544
SGOT	100 ± 12.2	84.8 ± 18.2	<0.001
SGPT	79.3 ± 14.2	71.3 ± 16.2	0.003
ESR	27.6 ± 8.2	27.2 ± 7.2	0.776
Neutrophil (%)	79.5 ± 3.5	66.2 ± 4.7	<0.001

Hepatic enzyme concentrations also showed significant differences, with SGOT levels being higher in non-eosinophilic patients (100 ± 12.2 U/L) compared to eosinophilic patients (84.8 ± 18.2 U/L, p<0.001, t = 4.21), and SGPT levels similarly elevated (79.3 ± 14.2 U/L versus 71.3 ± 16.2 U/L, p=0.003, t = 2.15). Neutrophil percentages were significantly higher in the non-eosinophilic group (79.5 ± 3.5%) compared to the eosinophilic group (66.2 ± 4.7%, p<0.001, t = 12.84), further supporting the concept of different inflammatory patterns between the phenotypes.

Analysis of arterial blood gases offered further understanding of breathing conditions in the two subgroups, as outlined in Table 3. The non-eosinophilic cohort exhibited a notably elevated average pH value (7.34 ± 0.11) relative to the eosinophilic cohort (7.30 ± 0.12, p=0.005), pointing to distinct forms of breathing distress and pH equilibrium. Additional blood gas metrics, such as CO2 partial pressure, O2 partial pressure, bicarbonate amounts, and electrolyte readings, displayed no meaningful statistical variances between subgroups, although patterns hinted at diverse extents of lung dysfunction.

**Table 3:** ABG analysis of AECOPD patients AECOPD: Acute exacerbation of chronic obstructive pulmonary disease; ABG: arterial blood gas

ABG Parameters	Non-eosinophilic (AEC ≤300 cells/μL) (n=95)	Eosinophilic (AEC >300 cells/μL) (n=47)	p-value
pH	7.34 ± 0.11	7.30 ± 0.12	0.005
PaCO2	46.11 ± 11.5	44.74 ± 12.2	0.513
PaO2	91.21 ± 22.3	89.6 ± 26.3	0.703
HCO3	31.2 ± 9.2	29.5 ± 8.5	0.290
Serum Na	140.5 ± 18.0	138.7 ± 19.8	0.588
Serum K	3.89 ± 0.91	3.62 ± 0.92	0.099

**Figure 2:**
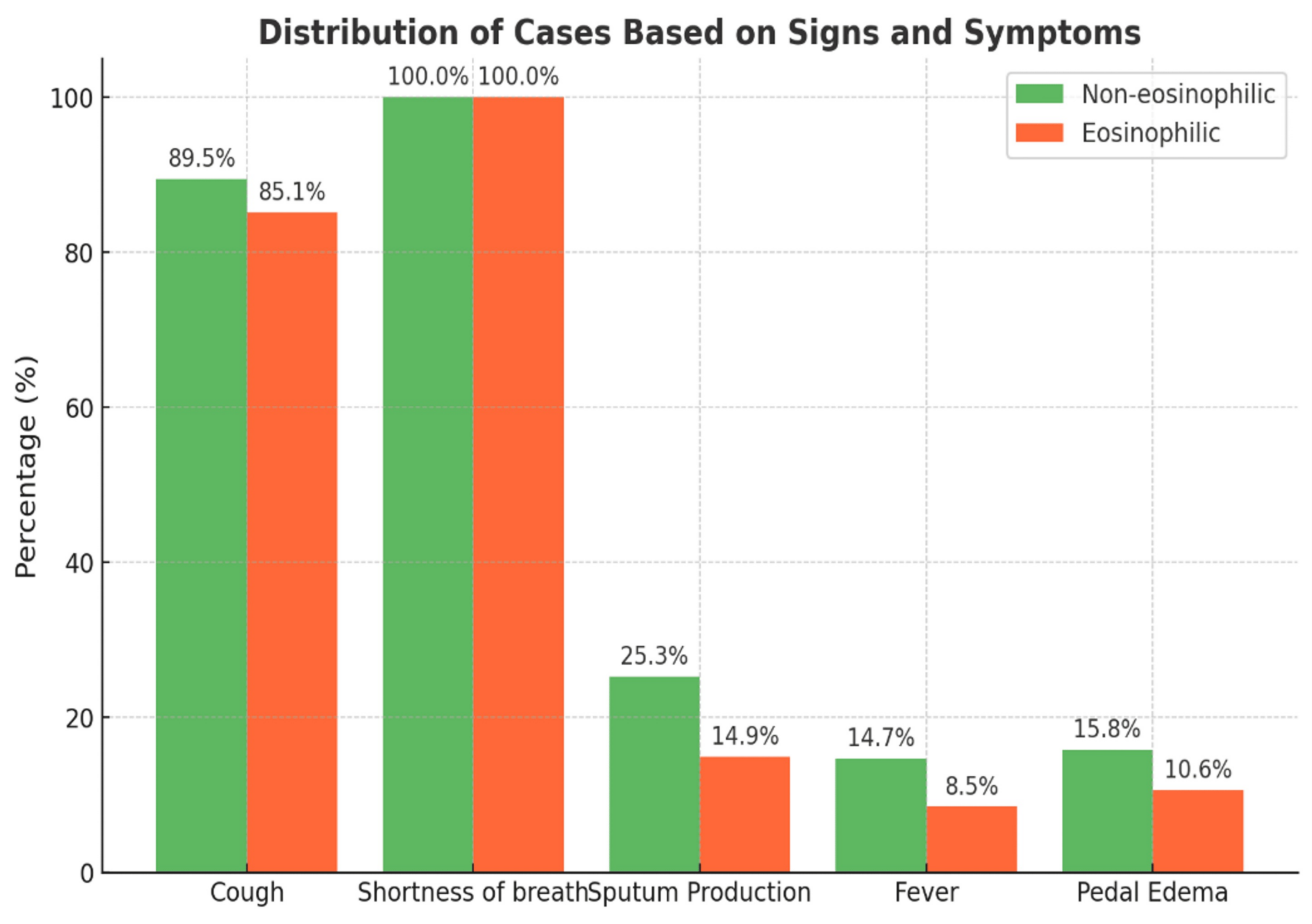
Distribution of cases based on sign and symptoms

Clinical outcomes analysis revealed important differences in hospital course and treatment requirements between the two phenotypes, as illustrated in Figure 2. The eosinophilic group demonstrated a shorter median hospital stay (four days) compared to the non-eosinophilic group (six days, p=0.002), suggesting better clinical outcomes and faster recovery in eosinophilic patients. Although respiratory failure, bilevel positive airway pressure requirement, and mortality rates were numerically higher in non-eosinophilic patients, these differences did not reach statistical significance, though the trends suggest a more severe disease course in non-eosinophilic COPD.

Multivariable logistic regression was conducted to pinpoint predictors of respiratory failure among individuals with COPD, with outcomes displayed in Table 4. Results suggested that advanced age (over 70 years) was associated with increased likelihood of respiratory failure (OR 5.333, 95% CI 1.443-19.00, p=0.012). Being female exhibited a notable connection to respiratory failure (OR 2.100, 95% CI 1.0537-4.1854, p=0.035). Tobacco use history displayed the most potent correlation with respiratory failure (OR 69.0909, 95% CI 15.4444-309.07, p<0.001). Manifestations such as coughing (OR 6.4925, 95% CI 1.446-29.5885, p=0.015) and mucus expectoration (OR 23.0417, 95% CI 6.5389-81.1933, p<0.001) were likewise strongly tied to greater chances of respiratory failure.

**Table 4:** Results of multivariate logistic regression analysis of factors associated with respiratory failure in patients Data presented as number (N) and percentage (%). Odds ratios with 95% confidence intervals calculated using binary logistic regression. P<0.05 considered statistically significant. AEC: Absolute eosinophil count; SGOT: serum glutamic-oxaloacetic transaminase; SGPT: serum glutamate pyruvate transaminase

Variables		Respiratory Failure (n=60)	Non-respiratory Failure (n=82)	Odds Ratio	95% CI	p-value
AEC	Non-eosinophilic	43	52	1.2767	0.6252 – 2.6073	0.502
	Eosinophilic	17	30	Reference category		
Age (years)	≤ 50 years	5	12	Reference category		
	50 – 70 years	35	61	1.3770	0.4479 – 4.233	0.576
	> 70 years	20	9	5.333	1.443 – 19.00	0.012
Gender	Male	40	40	Reference category		
	Female	20	42	2.100	1.0537 – 4.1854	0.035
Smoking	No	22	80	Reference category		
	Yes	38	2	69.0909	15.4444 – 309.07	<0.001
Cough	No	2	15	Reference category		
	Yes	58	67	6.4925	1.446 – 29.5885	0.015
Sputum Production	No	28	79	Reference category		
	Yes	32	3	23.0417	6.5389 – 81.1933	<0.001
CRP (mg/L)	3 to 10	22	39	Reference category		
	> 10	38	43	1.566	0.7930 – 3.0949	0.196
SGPT (U/L)	7 to 56	41	47	Reference category		
	> 56	19	35	1.606	0.7995 – 3.2299	0.183
SGOT (U/L)	5 to 40	14	19	Reference category		
	> 40	46	63	1.009	0.4589 – 2.2193	0.981
pH	7.35 to 7.45	30	40	Reference category		
	< 7.35	30	42	1.050	0.539 – 2.0438	0.885
Neutrophil (%)	60 to 70	20	29	Reference category		
	> 70	40	53	1.094	0.5422 – 2.208	0.801

Examination of therapeutic approaches uncovered notable links between eosinophil levels and steroid administration while in the hospital, as illustrated in Table 5. Those with eosinophilic characteristics were more prone to being prescribed inhaled steroids (66.0%) than individuals with reduced eosinophil numbers. On the other hand, intravenous or oral steroids were administered more often to those with extremely low eosinophil values (<150 cells/μL), affecting 46.7% of them versus just 19.1% of the eosinophilic group. This association was statistically significant (χ² = 11.332, p = 0.023), underscoring the importance of using eosinophil metrics to guide therapy choices.

**Table 5:** Association between corticosteroid use and eosinophil count in hospitalized COPD patients Statistical significance determined by chi-square test. p<0.05 considered statistically significant.

Corticosteroid Use During Hospitalization	Eosinophil Count			χ² = 11.332 p-value = 0.023
	<150 (n=45)	150-300 (n=50)	>300 (n=47)	
None	4 (8.9%)	6 (12.0%)	7 (14.9%)	
Only Inhaled Corticosteroid	20 (44.4%)	34 (68.0%)	31 (66.0%)	
Systemic Corticosteroid	21 (46.7%)	10 (20.0%)	9 (19.1%)	

## Discussion

The findings from this comprehensive study provide valuable insights into the distinct characteristics of eosinophilic and non-eosinophilic COPD phenotypes during acute exacerbations, supporting the growing evidence for phenotype-based management approaches in COPD care. The observed prevalence distribution, with 66.9% of patients having non-eosinophilic COPD and 33.1% having eosinophilic COPD, aligns with existing literature while reflecting some regional variations in phenotype distribution [14].

The demographic differences observed between the two phenotypes provide important clinical insights. The tendency for eosinophilic patients to be younger (mean age 57.81 years) compared to non-eosinophilic patients (mean age 62.94 years) has been consistently reported in previous studies and may reflect different disease progression patterns or underlying pathophysiological mechanisms [15]. The slight female predominance in the eosinophilic group (51.06%) contrasts with the male predominance in the non-eosinophilic group (60.0%), suggesting potential gender-related influences on inflammatory phenotype development that warrant further investigation [16]. The striking difference in smoking prevalence between the groups, with non-eosinophilic patients showing higher smoking rates (33.7% versus 17.0%), supports the hypothesis that different environmental and genetic factors may contribute to phenotype development. The majority of patients in both groups are non-smokers reflects the increasing recognition of COPD in never-smokers, particularly in regions with high levels of environmental pollution and biomass fuel exposure [17].

The laboratory findings represent the most compelling evidence for distinct pathophysiological mechanisms underlying these phenotypes. The significantly elevated C-reactive protein levels in non-eosinophilic patients (17.35 ± 3.8 mg/L versus 8.2 ± 5.3 mg/L, p<0.001) indicate a more pronounced systemic inflammatory response, which may contribute to the observed differences in clinical outcomes and treatment responses [18]. This finding is consistent with the established understanding that CRP serves as a reliable marker of systemic inflammation and has prognostic value in COPD exacerbations. The elevation of hepatic enzymes (SGOT and SGPT) in non-eosinophilic patients suggests either systemic effects of increased inflammation or potential medication-related hepatotoxicity, though the latter seems less likely given the acute presentation. The significantly higher neutrophil percentages in non-eosinophilic patients (79.5% versus 66.2%, p<0.001) further support the concept of different inflammatory cascades, with neutrophilic inflammation being more prominent in the non-eosinophilic phenotype [19].

The arterial blood gas analysis, revealing higher pH levels in non-eosinophilic patients, while counterintuitive given their apparently more severe inflammatory profile, may reflect compensatory mechanisms or different patterns of ventilatory response to acute exacerbations. This finding requires further investigation to understand the underlying physiological mechanisms and their clinical implications [20]. The clinical outcomes analysis provides crucial evidence for the prognostic value of eosinophil-based phenotyping. The shorter hospital stay in eosinophilic patients (4 versus 6 days, p=0.002) supports previous reports of better short-term prognosis in eosinophilic COPD exacerbations. This difference likely reflects the distinct inflammatory mechanisms and potentially better treatment responsiveness in eosinophilic patients, particularly to corticosteroid therapy [21].

The multivariate logistic regression analysis identifying risk factors for respiratory failure provides clinically actionable insights. The strong association between smoking history and respiratory failure (odds ratio 69.09) emphasizes the continued importance of smoking cessation in COPD management. The association of older age, female gender, and specific symptoms with respiratory failure risk can inform clinical decision-making and risk stratification strategies [22].

Perhaps most importantly, the significant association between eosinophil count and corticosteroid use patterns (p=0.023) validates current trends toward personalized treatment approaches. The preferential use of inhaled corticosteroids in eosinophilic patients and systemic corticosteroids in patients with very low eosinophil counts reflects emerging evidence-based treatment paradigms. This finding supports the potential for eosinophil-guided therapy to optimize treatment efficacy while minimizing unnecessary medication exposure and associated side effects [23]. The implications of these findings extend beyond the immediate clinical care of individual patients to broader healthcare system considerations. The ability to identify patients likely to benefit from specific treatments based on readily available biomarkers could significantly improve resource allocation and cost-effectiveness of COPD care. Additionally, the recognition of distinct phenotypes may inform future drug development efforts and clinical trial design [24].

Several important limitations must be acknowledged when interpreting these findings. The cross-sectional design captures only a single time point during acute exacerbations and cannot establish causal relationships between eosinophil phenotypes and observed clinical differences. Potential confounding factors, including prior medication use (particularly corticosteroids and antibiotics), comorbid conditions, environmental exposures, and varying severity of baseline COPD, were not fully controlled for in this analysis. The single-center design may limit generalizability to other populations with different demographic characteristics or environmental exposures. Additionally, the convenience sampling approach and lack of formal power calculation represent methodological limitations that may affect the precision of our estimates.

## Conclusions

This study demonstrates significant associations between eosinophil phenotypes and clinical characteristics in COPD patients during acute exacerbations. Non-eosinophilic patients showed associations with more severe systemic inflammation, including elevated C-reactive protein, higher neutrophil counts, increased hepatic enzymes, and longer hospital stays, suggesting a potentially more aggressive disease phenotype. Eosinophilic patients were associated with better short-term outcomes and a higher likelihood of receiving targeted inhaled corticosteroid therapy.

The observed associations between eosinophil count and corticosteroid use patterns support the potential for eosinophil-guided treatment approaches, though these findings are hypothesis-generating rather than definitive. The distinct inflammatory profiles suggest that different phenotypes may benefit from tailored therapeutic strategies, supporting the movement toward precision medicine frameworks in COPD management. These findings reinforce the clinical utility of COPD phenotyping and suggest that measuring blood eosinophils during exacerbations could potentially enhance treatment guidance. However, prospective, multicenter studies with longer follow-up periods are needed to confirm these associations, establish causality, and evaluate the long-term implications of phenotype-specific interventions on COPD management and patient outcomes.
